# Advanced Therapeutics in Parkinson’s Disease: What’s New?

**DOI:** 10.1007/s11910-026-01483-5

**Published:** 2026-02-18

**Authors:** Mariana H.G. Monje, Jessica A. Karl, Christa S. Cooper, Jacob Yomtoob, Juan Deliz, Lucy A. Morse, Neil Shetty, Leonard Verhagen Metman

**Affiliations:** Ken and Ruth Davee Department of Neurology, Feinberg School of Medicine, Northwestern University, Lavin Family Pavilion, 259 E Erie St suite 1900, Chicago, IL 60611 USA

**Keywords:** Infusions, Deep brain stimulation, Interventional magnetic resonance imaging, High-intensity focused ultrasound ablation, Cell therapy

## Abstract

**Purpose of Review:**

To provide a concise, clinically oriented update on advanced therapies for Parkinson’s disease.

**Recent Findings:**

Subcutaneous infusion of foscarbidopa/foslevodopa and apomorphine reduce OFF time and improve “good ON time”, though infusion site reactions remain of some concern. Image-guided programming may shorten programming time while matching motor outcomes; while adaptive/closed-loop deep brain stimulation using local field potential signals may improve symptoms and quality of life with a lower energy use. Remote programming accelerates clinical benefit and expands access. Approved magnetic resonance guided high intensity focused ultrasound targets now include the ventralis intermediate nucleus of the thalamus, the globus pallidus pars interna and, recently, the pallidothalamic tract; while research investigates the subthalamic nucleus, each target with distinct benefits and adverse event profiles. Early studies using magnetic resonance guided low intensity focused ultrasound show safe, transient blood brain barrier opening. First-in-human stem cells-derived dopaminergic grafts show safety and graft functioning.

**Summary:**

The advanced therapeutic landscape for Parkinson’s disease has evolved through innovations in established and novel therapies. Future priorities for the field include standardized biomarkers and protocols for adaptive deep brain stimulation, long-term evaluation of high intensity focused ultrasound outcomes, and rigorously controlled trials of low intensity focused ultrasound and cell-based therapies designed to assess disease-modifying potential.

## Introduction

In recent years, the therapeutic landscape for advanced Parkinson’s disease (PD) has evolved through significant innovations in established or new device-based and other advanced therapies. Continuous dopaminergic stimulation can now be achieved not only via intestinal infusion, but also with novel subcutaneous infusion systems, offering an effective and less invasive treatment option for motor fluctuations. Deep brain stimulation (DBS) remains a key therapy for advanced PD, with ongoing refinements in targeting, programming, and with new and potentially useful features such as sensing and adaptive stimulation. Magnetic resonance guided focused ultrasound (MRgFUS) has emerged as an incisionless surgical option for select PD patients, with the number of targets continuously expanding. Meanwhile, new early-phase clinical trials of cell-based therapies are underway, aiming to restore dopaminergic function through grafting strategies, marking a return to the field’s long-standing goal of circuit-level repair.

In this review, we summarize key therapeutic advances over the past few years in advanced interventional therapies for PD—including pumps, DBS, MRgFUS, and cell-based approaches, focusing on innovations that have shaped or redefined the role of these state-of-the-art interventions. This is not a systematic review, and we readily acknowledge that the selection of topics is incomplete and subject to our collective biases. However, we used author consensus and a consistent approach to select and appraise the most relevant developments.

## Continuous Infusion Therapies

Motor fluctuations arise in part due to the non-physiological, pulsatile stimulation of dopamine receptors caused by intermittent oral levodopa dosing. In contrast, continuous dopaminergic stimulation (CDS) aims to provide a steadier dopaminergic tone, mirroring the tonic firing of healthy nigrostriatal neurons and reducing motor fluctuations. Continuous infusion therapies are non-oral treatment alternatives that use drug delivery systems that bypass the gastrointestinal tract with improved bioavailability and steadier plasma drug levels. In advanced disease, these non-oral systems have demonstrated meaningful reductions in OFF time and dyskinesias, leading to increased “good ON” time. Currently available strategies include therapies that take advantage of intestinal and subcutaneous (SQ) routes of administration.

### Intestinal Infusions

Levodopa carbidopa intestinal gel (LCIG) is a stable gel suspension of levodopa-carbidopa for continuous infusion typically via a percutaneous endoscopic gastrostomy with jejunal extension [1]. The therapy was first available in Sweden in 2004 and achieved the U.S. Food and Drug Administration (FDA) approval in 2015. Initial studies into LCIG demonstrated benefit in terms of reduction in OFF time, increases in ON time without troublesome dyskinesias and improvements in quality of life and activities of daily living [2–6]. More recently, results from the international and observational DUOGLOBE prospective registry (*N* = 195) demonstrated significant and maintained improvements in OFF time over the span of 36 months [7] (Table 1). Additionally, there were also sustained improvements in dyskinesias, non-motor symptom burden, sleep, daytime sleepiness, health-related quality-of-life and caregiver burden. About 16% of participants who experienced some serious adverse events (AEs) were deemed to have a reasonable possibility of causal relationship to LCIG. There was one death with possible relation to the treatment due to intestinal obstruction in a patient with a medical history of diverticulitis. The study was limited by a high study discontinuation rate of 50%, though notably 30% of those individuals who stopped participating in the study continued to use LCIG outside the study, supporting the long-term effectiveness of the therapy.

**Table 1:** High-Level comparison of advanced therapies for parkinson’s disease: regulatory status of advanced PD therapies in the US and Europe. This table provides a regulatory overview for each therapy in the US and Europe, supporting clinical and practical decision-making

Therapy	Indications	Regulatory Status		Key Advantages	Major Limitations/Concerns	Ref
		US	Europe			
Levodopa-Carbidopa Intestinal Gel (LCIG)	Motor fluctuations in adults with advanced PD.	FDA approved (Duopa^TM,^ 2015)	EMA approved (Duodopa^®^,2004)	Continuous delivery, robust efficacy, improves ON time, reversible	Requires surgery (PEG tube), device complications, infection risk	[1–6]
Levodopa-Entacapone-Carbidopa Intestinal Gel (LECIG)	Advanced PD with severe motor fluctuations	Not approved	EMA approved (Lecigon^®^, 2019)	Continuous delivery, lower levodopa dose, smaller pump, compatible with existing PEG-J, reversible	Same as LCIG Newer product, long-term data limited	[8–10]
Subcutaneous Foslevodopa/Foscarbidopa Infusion	Motor fluctuations in adults with advanced PD.	FDA approved (Vyalev™, 2024)	EMA approved (Produodopa ^®^, 2022)	Continuous delivery, improves ON time, reduces OFF time, 24 h delivery, reversible	Infusion site reactions, long-term tolerability unknown, device management, adherence	[11–15]
Subcutaneous Apomorphine Infusion (CSAI)	Motor fluctuations in adults with advanced PD.	FDA approved (Onapgo™, 2024)	EMA approved, 1993 (Apo-Go^®^, Apomorhonine Hydrochloride)	Adjuvant therapy, continuous delivery, improves ON time, reduces OFF time, reversible	Infusion site reactions, device management, adherence	[16–20]
Deep Brain Stimulation (DBS)	– Tremor predominant PD (VIM) – Levodopa-responsive PD of at least 4 years’ duration that are not adequately controlled with medication, including motor complications of recent onset (from 4 months to 3 years) or motor complications of longer-standing duration (STN, GPi)	FDA approved for PD VIM: 1997 GPi: 2002 STN:2002	CE marked and widely used for PD (STN, GPi, VIM), 1990s)	Robust motor benefit, adjustable, reversible, STN-DBS significant levodopa sparing, long-term data available	Invasive surgery, perioperative risks, not for dementia/elderly, target-dependent symptom control	[21–24]
Closed-Loop/ Adaptive DBS	Approval is under the existing DBS indication, with adaptive stimulation allowed as a commercial clinical mode.	FDA approved (Medtronic BrainSense™ AdaptiveDBS (Percept™ platform), 2025)	CE marked approved (Medtronic BrainSense™ AdaptiveDBS (Percept™ platform), 2025)	Possibly improved benefit/side effect profile, lower energy use, personalized therapy	Limited availability, time consuming, long-term data pending	[25–33]
MR-guided high intensity focused ultrasound (MRgHIFU)	– Tremor-dominant PD with medication-refractory tremor (VIM) – Medication-refractory moderate to severe motor complications as an adjunct to medication (GPi) – Medication-refractory moderate to severe motor complications as an adjunct to medication treatment, including staged contralateral unilateral procedures performed at least 6 months after the initial treatment. (PTT) -Global motor symptoms improvement (STN)	FDA approved for PD, for unilateral VIM 2018; GPi 2023; PTT (also staged) 2025 STN: investigational	CE marked for VIM 2018, GPi 2023, and STN 2022 (limited)	Incisionless, outpatient, no hardware, expanding targets, alternative for non-DBS candidates	Irreversible, target-dependent symptom control, target-specific risks, limited long-term data	[34–39]
MR-guided low intensity focused ultrasound (MRgLIFU)	Investigational for targeted blood–brain barrier opening for drug delivery and neuromodulation	Experimental/clinical trials only	Experimental/clinical trials only	Potential for disease modification, promising safety and feasibility	Only in trials, long-term efficacy/safety unknown	[40–42]
Cell-based Therapies	Investigational for cell replacement (dopaminergic progenitor-derived neurons)	Experimental/clinical trials only	Experimental/clinical trials only	Potential for long-term neurorestorative effect and disease modification, early safety demonstrated	Only in trials, long-term efficacy/safety unknown, immunosuppression	[43–46]

Levodopa-entacapone-carbidopa intestinal gel (LECIG) was first introduced in Sweden in 2018 to further increase the bioavailability of LCIG and minimize total daily levodopa requirements and infusion volumes [8] (Table 1). The first retrospective report on the long-term clinical use of LECIG infusion was published in 2024 [9]. The study followed 24 patients with advanced PD on LECIG therapy over a 4-year period. 21% discontinued LECIG due to side effects (most frequently diarrhea) and 8/24 patients died while receiving LECIG, though causal association with LECIG was not directly addressed. The ongoing observational ELEGANCE study is gathering long-term efficacy, safety and patient-reported outcomes with LECIG [10]. Interim analysis of this cohort showed findings consistent with prior reports with sustained reduction in OFF time of > 3 h/day up to 1 year of treatment and additional benefits on sleep and quality of life measures.

### Subcutaneous Infusions

Foscarbidopa/foslevodopa (FCD/FLD, ABBV-951) was recently introduced and FDA approved in 2024 as a viable phosphate prodrug of carbidopa/levodopa (CD/LD) for continuous SQ infusion via a portable pump over 72 h [11, 12]. In a 12-week, double-blind, active-controlled, phase 3 trial (*N* = 141), FCD/FLD demonstrated significant improvement in ON time without troublesome dyskinesias (2.72 active vs. 0.97 oral), OFF time and morning akinesia when compared to oral CD/LD [13]. The most frequent AEs in the FCD/FLD group involved infusion site events (72%, including erythema, pain, cellulitis and edema) (Table 1). Other less common infusion site AEs included bruising, hemorrhage, nodules, induration, and pruritus. Most of these reactions were non-life threatening, but up to moderate in severity. Of note, AEs led to premature discontinuation of FCD/FLD infusion in 22% of participants in the treatment group [13]. In a larger (*N* = 244), open-label, 52-week, phase 3 study, mild-to-moderate infusion site events were again the most common AEs [14]. AEs were reported in about 26% of participants, with infection site cellulitis and abscess being the most common. Out of 107 participants who discontinued the treatment 26.2% had AEs contributing to its discontinuation, most commonly during the initial titration period [14]. These observations along with real-world experience have led to algorithms for the management of infusion site events to limit interrupting SQ therapy [15]. An alternative formulation of subcutaneous CD/LD (i.e. ND0612) demonstrated a similar promising benefit-risk profile [47]. Nonetheless, the ND0612 delivery system can only administer 720 mg/day of levodopa and can therefore not completely replace oral levodopa in those individuals with a higher daily levodopa requirement.

The therapeutic use of continuous SQ apomorphine infusion (CSAI) in the management of PD has long been recognized in Europe for decades, though few randomized controlled trials have been performed [16]. The European TOLEDO 12-week, randomized controlled trial and open-label extension provided level I evidence for the safety and efficacy of CSAI in addressing motor fluctuations in patients with PD, with sustained benefit up to 52 weeks [17, 18]. Concurrently, the prospective, open-label, phase 3 InfusON trial was initiated in the US to evaluate safety, tolerability and efficacy of CSAI [19]. In the latter study, patients on CSAI experienced reduced OFF time (by 3.15 h/day) with improved good ON time (by 3.70 h/day) at the 52-week endpoint of the study. CSAI was used as an adjunctive to optimized oral dopaminergic therapies, which represents an alternative management strategy for continuous infusion therapies [19] (Table 1).

The utilization of CSAI has been mainly studied for diurnal use. However, in the recent APOMORPHEE trial, participants with advanced PD with moderate-to-severe insomnia (*N* = 46) were randomized to nighttime-only CSAI or placebo. Those in the CSAI group experienced significant self-reported improvement in sleep disturbances, with most notable improvements in overall sleep quality, sleep onset and maintenance insomnia [20].

In summary, CDS, in contrast to pulsatile oral dopaminergic delivery, has been shown to effectively treat motor fluctuations and reduce the severity of dyskinesias in PD. Although it has been hypothesized that early initiation of CDS could prevent the development of motor response complications, clinical evidence to support this remains limited, primarily due to the lack of suitable, non-invasive therapeutic options for newly diagnosed patients [48].

## Deep Brain Stimulation

DBS is a well-established and widely used therapy for advanced PD [21–24] (Table 1). Recent advances in DBS include image-guided programming using volume of tissue activation (VTA) models, sensing of local field potentials (LFP) to identify optimal contacts, adaptive stimulation to potentially improve outcomes and reduce side effects, and remote programming to enhance access for patients with travel limitations. Emerging strategies, such as early DBS for potential disease modification, multitarget stimulation, and novel targets, illustrate the expanding scope of the field.

### Imaged Guided Programing

Conventional DBS programming using a standard mono-polar survey to identify therapeutic motor benefit and side effects at each electrode remains the gold-standard for selecting stimulation settings [49]. However, this process is time-consuming and has become increasingly complex with the advent of directional lead technology. To assist in this process, several visualization software systems have been developed, including Boston Scientific Guide XT/STIMVIEW XT (in partnership with Brainlab), Brainlab Elements, and LEAD-DBS (MATLAB) [50]. Image guided programming (IGP) leverages patient-specific anatomical mapping, lead and electrode localization, and modeling of the volume of tissue activated (VTA) by DBS settings.

Several preliminary studies have demonstrated that IGP strongly correlates with the optimal clinically selected omni-directional ring stimulation setting up to one-year post-DBS and significantly reduces programming time [49–53]. In some cases, there was less concordance in the selection of directional electrodes; however, this was primarily because patients were not clinically programmed with directional electrodes, likely due to time constraints [49, 52]. In a study by Torres et al. (2024) patients with a suboptimal response to DBS were reprogrammed with IGP [54]. As a result, 37% of these patients were switched to directional stimulation, leading to an improvement in motor symptoms, as measured by 5-point (22%) reduction in the MDS-UPDRS Part III score, highlighting the clinical benefits of IGP. Several approaches have been proposed for utilizing IGP in its current form, including: (1) using IGP to identify the two most optimal electrode levels, followed by directional testing with a standard monopolar survey; and (2) using IGP for initial electrode level selection, with subsequent clinical programming to refine the settings [52, 55].

In summary, IGP demonstrated high inter-rater reliability in selecting the optimal electrode level, provided motor outcomes that were non-inferior to clinical programming, and required less time. Potential challenges associated with IGP include: (1) logistical difficulties related to image processing and anatomical model construction, (2) the need for precise anatomical mapping and lead localization, and (3) limitations in biophysical model accuracy. Larger, randomized, multicenter studies are necessary to validate these preliminary findings.

### Closed-Loop/Adaptive DBS

Conventional DBS (cDBS) delivers continuous stimulation using fixed, clinician-programmed settings and does not account for changes in motor state or underlying neural activity. Building on the established spatial precision of cDBS, the next evolution in DBS introduces temporal adaptability through sensing-enabled, adaptive systems that adjust stimulation in response to real-time physiological feedback. These technologies aim to further optimise clinical outcomes by aligning therapy delivery with moment-to-moment changes in neural activity.

Sensing-enabled DBS refers to DBS systems capable of recording physiological signals, most commonly local field potentials (LFPs) from implanted electrodes, without necessarily modifying stimulation. Closed-loop (clDBS) and adaptive deep brain stimulation (aDBS) are terms often used interchangeably to describe a DBS system which dynamically modulates stimulation parameters in real time based on physiological feedback signals, such as the aforementioned LFPs. The term *closed-loop* emphasises the feed-back nature of the system—sensing neural activity and responding to it—whereas *adaptive* emphasises the dynamic (continuous or state-dependent) adjustments of stimulation parameters. For clarity and consistency, the term aDBS is used throughout this manuscript. Therefore, in contrast to cDBS, aDBS enables real-time parameter adjustment in response to ongoing biofeedback. This approach holds the potential to enhance symptom control, minimize side-effects, and reduce energy consumption. Case reports have suggested clinical advantages of aDBS over cDBS, including improved gait by avoiding overstimulation [25], reduced bradykinesia and enhanced quality of life [26], and better motor symptom control with and without dopaminergic medications [27]. However, individualized tuning is essential for optimizing patient outcomes (Table 1).

LFPs, particularly beta-band frequencies within the target nuclei, are commonly utilized as biomarkers in aDBS. Longer beta burst activity is associated with increased motor impairment, whereas shorter-duration and lower-amplitude beta bursts are correlated with improved motor function [28]. Low-beta activity (13–20 Hz) tends to decrease with increasing stimulation, a change that correlates with improved bradykinesia [29]. LFPs are reliable biomarkers for aDBS in both on- and off-medication states in PD [30]. However, small studies suggest that even minor deviations from the selected feedback frequency can compromise therapeutic efficacy. Therefore, patient-specific beta activity profiles and personalized parameter adjustments are critical for achieving optimal results [31, 32]. Additionally, improved rigidity has been linked to the proximity of aDBS contacts to spectral “hot spots” corresponding to therapeutic frequency bands [33]. The success of aDBS depends heavily on the chosen control frequency and the accurate selection of active electrode contacts. Despite promising results, there are currently no standardized protocols for aDBS implementation in PD. Given the numerous factors influencing clinical outcomes, the development of standardized guidelines is essential to ensure consistent and effective applications of aDBS in practice [56].

Emerging concepts in aDBS include the use of gamma oscillations in addition to beta frequencies, sleep stages as biomarkers, and a technique known as evoked interference DBS (eiDBS) targeting the globus pallidus internus (GPi). eiDBS delivers precisely timed electrical impulses in response to GPi LFPs, effectively modulating neural activity with minimal power amplitudes [57]. A small crossover study found gamma oscillations to be a reliable marker of motor fluctuations and dopaminergic state and, when used as a biofeedback signal for aDBS, it outperformed cDBS for improvements in motor symptoms and quality of life [58]. Narrowband gamma oscillations (65–90 Hz) in the STN or motor cortex correlated with the onset of dyskinesia in real time, without detectable lag, supporting their use as a potential biomarker for aDBS [59]. Sleep dysfunction is a frequent and debilitating symptom in PD. A proof-of-concept study demonstrated the feasibility of using sleep stages, particularly non-REM stage 3, as a biomarker for aDBS, offering a potential pathway for targeted neuromodulation to treat sleep-related symptoms [60]. However, larger clinical trials are needed to validate these approaches and establish their clinical utility. In February 2025, the FDA approved Medtronic’s Percept neurostimulator, which incorporates BrainSense™ Technology, as the first aDBS device for PD [61].

While LFPs have been getting the most attention, as they are obtained from the pathologically oscillating circuits using electrodes already in place, peripheral feedback sources, such as muscle electromyography (EMG) or wearable devices, have also been explored for use in aDBS systems. In one study, an EMG-driven model successfully modulated stimulation amplitude and pulse duration to effectively control tremor and beta activity with reduced power delivery compared to cDBS [62]. While tremor suppression was similar between aDBS and cDBS in a trial utilizing a smartwatch [63], another trial using a Kinesia device found that cDBS provided superior control of rigidity and finger tapping [64]. These wearable-enabled approaches may reduce the need for continuous clinician oversight, offering potential benefits for patients in remote locations or underserved areas [63, 64].

### Remote DBS Programming

Whether in remote areas or not, patients often struggle to gain access to DBS centers. Remote programming represents a key step towards increasing accessibility of DBS therapy, enabling clinicians to adjust settings beyond the confines of in-person visits.

Since its FDA approval in 1997, patients with DBS have been required to visit a specialist in person for device checks and programming. This presents challenges due to the frequency of visits, the limited number of specialized centers nationwide, and the resulting financial and time burdens on caregivers. In-person visits can also be uncomfortable and risky for patients, as DBS programming often requires them to pause their PD medications, an issue that becomes more problematic as the disease progresses and fall risk increases. These challenges were further amplified during the COVID-19 pandemic. In 2020, Abbott received FDA approval for remote DBS programming via telemedicine. Using the Neurosphere™ Virtual Clinic, clinicians can remotely connect to a patient’s internal pulse generator (IPG) via a secure, web-based portal, to conduct video consultations and adjust stimulation settings in real time. This technology is particularly valuable for patients who have difficulty accessing specialized DBS centers for ongoing management [65].

A human factors validation study demonstrated that remote DBS programming is both safe and effective [66]. In a prospective, multicenter, randomized, controlled trial, patients with PD who received remote programming experienced clinical benefit 15.1 days earlier than those who received conventional in-person programming (*p* = 0.022). This earlier benefit is believed to result from improved access to timely DBS setting adjustments. Additionally, the majority of patients (84%) reported that the remote programming platform was easy to use, and clinicians found it comparable to in-person sessions in 88% of cases [67]. Despite its advantages, remote DBS programming faces challenges that may limit widespread adoption. These include variability in patient access to a reliable internet connection and regulatory or reimbursement barriers across different healthcare systems [68]. Furthermore, only one of the DBS systems that are available in the United States offers a remote programming option, and not all DBS centers currently support teleprogramming infrastructure, limiting its use to select device manufacturers and institutions.

### Expanding the Scope of DBS: Beyond Symptom Control

DBS effectively reduces motor fluctuations and dyskinesias in patients with moderate to advanced PD [23]. Regulatory guidelines currently recommend its use in individuals with a disease duration of at least four years. Although DBS is generally not considered to modify the underlying pathophysiological process in humans, this view is not unchallenged. Preclinical studies suggest that subthalamic nucleus (STN) DBS may have disease-modifying effects if initiated earlier, as striatal dopaminergic denervation typically exceeds 90% around four years after diagnosis [69]. These findings have led to the growing interest in the potential benefits of earlier DBS intervention. Notably, studies on early DBS do not assess neuroprotection per se but rather aim to evaluate disease modification [70].

In a prospective, randomized, single-blind clinical trial (*N* = 29), Hacker et al. (2020) demonstrated that STN DBS implanted in the early stages of PD reduced the risk of disease progression, specifically development or worsening of dyskinesias and rest tremor severity at 5-years post-implantation compared to optimal medical therapy [71]. Additionally, patients in the early-DBS group required fewer medications, indicating reduced polypharmacy. Long-term follow-up at 11 years post-DBS continued to show fewer motor complications in the early-DBS group, with a between group difference of 3.5 points on the Unified Parkinson’s Disease Rating (UPDRS) Part IV scale (*p* = 0.03). However, this analysis was limited by a small sample size, with only 12 participants evaluated at that time point, four of whom had received DBS [72]. Dong et al. (2024) also investigated whether DBS influences disease progression but found no significant difference at two-year follow-up between patients treated with DBS and those receiving optimal medical therapy [73]. Notably, this study enrolled only patients with advanced PD (Hoehn and Yahr stage 2.5 and 3), which may have limited the ability to detect disease-modifying effects. A larger, multicenter trial led by Hacker et al. has been approved by the FDA and will be essential for validating the findings of these pilot studies (NCT00608717).

To better understand the variability in clinical response to DBS, Hacker et al. (2023) examined the relationship between stimulation volumes, fiber tract connectivity, and motor outcomes in a subset of patients (*N* = 14) from the early PD pilot trial [74]. The main findings were: (1) slower motor progression was associated with stimulation of cortical tracts originating from the supplementary motor area (SMA) and primary motor cortex (M1), but not the pre-SMA; (2) stimulation of fiber tracts corresponding to the clinical “sweet spot” was linked to slower motor progression; (3) patients with slower motor progression required lower dopaminergic medication doses and stimulation voltages; and (4) the optimal stimulation site in early PD patients was located slightly more ventrally and laterally than the typical target used in advanced PD. If validated in larger, prospective clinical trials, these findings could influence surgical planning and electrode placement strategies by incorporating individual connectivity profiles [74].

### Additional Considerations

In parallel with efforts to refine stimulation delivery and timing, other strategies have aimed to broaden the anatomical and symptomatic scope of DBS. These include multitarget approaches designed to leverage complementary circuit effects, as well as novel targets to address symptoms such as gait dysfunction, or cognitive decline, that often remain refractory to conventional DBS. Multitarget DBS approaches involve the simultaneous stimulation of multiple anatomical structures, such as the STN and the GPi. Multitarget approaches have been explored in the last decade in an attempt to achieve better symptom control [75, 76]. A synergistic effect of simultaneous STN and GPi DBS has been previously reported although it has not been studied systematically [75, 77, 78]. Recently, a prospective clinical trial explored the used of quadripolar DBS leads implanted in both the STN and GPi bilaterally connected to a single IPG that allowed all 16 contacts to be used for recording and stimulation [79]. Six patients with PD were studied over a 2-year period. Blinded evaluation of motor scores suggested that the combined effects of dual STN + GPi continuous DBS were superior to stimulation at either target al.one. This benefit was sustained at 2-year follow up with 98% improvement from baseline in the awake ‘ON time per day without troublesome dyskinesia’ and an 85% LEDD reduction. Future studies with larger numbers of patients should examine the potential of dual target STN + GPi continuous DBS.

## Focused Ultrasound

MR-guided high intensity focused ultrasound (MRgHIFU) has emerged as an incisionless interventional therapy for motor symptoms in PD patients. Following pivotal trials of unilateral VIM thalamotomy, subsequent studies have explored the use of additional targets, such as the GPi, pallidothalamic tract (PTT) and STN, as well as staged bilateral procedures. Additionally, MR-guided low-intensity focused ultrasound (MRgLIFU) is being explored for reversible neuromodulation and opening of the blood-brain barrier (BBB) to facilitate drug delivery in PD patients. Currently, only MRgHIFU has received FDA approval (Table 1).

### High Intensity Focused Ultrasound (HIFU)

Unilateral ventral intermediate nucleus (VIM) thalamotomy for tremor-dominant Parkinson’s disease (TDPD) received FDA approval in 2018 following a two-center double-blind randomized clinical trial (RCT) [34] (Table 1). Twenty patients with TDPD who underwent unilateral VIM MRgHIFU had significant improvement in tremor control relative to the sham procedure group at three months. On-medication median tremor scores improved 62% from a baseline of 17 points following FUS thalamotomy and 22% from a baseline of 23 points on the clinical rating scale for tremor after sham procedures. The most frequent AEs included paresthesias (66%) and ataxia (35%), which persisted after 12 months in 19% and 4% of the patients, respectively. A subset of patients (25%) reported non-disabling residual paresthesias at the 12-month mark, similar to what has been widely reported in VIM thalamotomy for essential tremor [80].

Longer-duration observations of unilateral VIM thalamotomy have since been undertaken, with several studies demonstrating sustained tremor benefit relative to baseline at 3–5-year timepoints [81–83]. However, some degree of tremor recurrence is common, with several studies reporting diminishing benefit in tremor control over time [81–83]. For example, one retrospective study reported that 23% of patients experienced some degree of recurrence during follow-up, ranging from 1 to 5 years [84]. Notably, targeting the VIM does not result in sustained improvement of other key symptoms of PD (i.e. bradykinesia and rigidity) [81] or in mood, anxiety, apathy, sleep and cognitive symptoms [85, 86]. Efforts to clarify predictors of tremor response and AEs profile suggest that tremor relapse may be associated with smaller lesions and younger age, with persistent adverse effects at 6 months being associated with larger lesions [87].

A key area of recent studies on the use of MRgHIFU surrounds exploration of non-VIM targets, including the GPi, the PTT and the STN (Table 1). Notably, unilateral MRgHIFU targeting the GPi was granted FDA approval in 2021 for the treatment of advanced PD symptoms. In a RCT (3:1, *n* = 94), unilateral GPi-MRgFUS improved motor function or reduced dyskinesia compared to sham procedure over a three-month period [35]. In this study, the primary outcome was defined as a decrease (improvement) of at least three points in the MDS-UPDRS part III, for the treated side in the off-medication state, or in the score on the Unified Dyskinesia Rating Scale (UDysRS) in the on-medication state. 29% of treated PD patients showed a ≥ 3-point improvement in MDS-UPDRS III (off-medication, treated side), 12% in UDysRS, and 28% on both scales. In contrast, among controls, 27% met only the MDS-UPDRS III criterion, none met only the UDysRS criterion, 5% met both, and 68% were classified as non-responders [35]. An anti-dyskinetic effect was observed in approximately 40% of patients [35]. Further trials and long-term data are needed to define its role and identify suitable candidates. PTT-MRgHIFU was applied unilaterally in a large open-label series [36] and bilaterally in an open, single-center study of 10 patients [88]. These studies demonstrated 70–80% improvement in tremor, rigidity and bradykinesia, as well as the additional benefit of suppressing dyskinesias. However, speech disturbance was a relatively common (15–20%) and sometimes sustained AEs [36]. A prospective, open label, single-arm, multicenter trial of bilateral staged PTT has recently been completed (NCT04728295) [89], with published results expected later this year. In July 2025, the FDA approved the unilateral and staged PPT-MRgHIFU treatment [37] (Table 1). Dual targeting of PTT and VIM has also been explored [90].

Additionally, over the last five years, several studies have been published using unilateral MRgHIFU targeting the STN to treat, in addition to tremor, bradykinesia and rigidity [38, 91, 92]. A RCT (2:1) was conducted in 40 markedly asymmetrical PD patients [38] (Table 1). Treated patients experienced clinically significant improvement in the MDS-UPDRS-III score of the treated hemibody compared to those who received a sham-procedure at 4 months; 19.9 ± 5.0 at baseline to 9.9 ± 4.9 at 4 months, as opposed to a decrease in the control group from 18.7 ± 5.5 at baseline to 17.1 ± 6.0 at 4 months. AEs were frequent but mostly mild and transient, including mild speech disturbance (48%) and gait disequilibrium (56%) and had fully resolved in all patients after one year. Of interest, six patients (22%) developed off-dyskinesia in the first week after the procedure, which persisted, although mildly, at 4 months in three patients (11%). Weakness on the treated side was present the day after active treatment in five patients (19%), which resolved within 4 weeks post-procedure in three patients and fully recovered at 12 months in the other two patients.

The long-term (36-month) follow-up of these patients demonstrated sustained benefit without delayed AEs [93]. In 32 patients reported, 26% developed nondisabling ON-dyskinesias at 36 months post-procedure, though none with persistent OFF dyskinesias, and none with disabling dyskinesias. Two AEs (hand clumsiness, dysarthria) were rated as moderate in severity at 36 months, though overall AE rates were low [39]. Similar findings have been reported in prospective open-label studies of unilateral STN MRgHIFU lesions showing significant motor improvement with over 50% reduction in tremor, rigidity and bradykinesia on the treated side, while gait and balance remained stable [91, 92]. Ultimately, the STN target offers additional and potentially sustained benefit for PD management, but the AE profile should be carefully considered. A recent retrospective two-center study aimed to compare unilateral VIM-MRgHIFU versus STN-MRgHIFU on TDPD, reporting greater tremor control at 12-month follow-up in the STN group, as well as greater benefit for bradykinesia and rigidity [94]. In line with other studies, AEs profiles differed, with greater frequency of gait related adverse effects in VIM targeted PD patients and worsening of dyskinesia exclusively observed in STN targeted PD patients [94]. In sum, the potential benefits of VIM versus STN targeting should be weighed against varying AEs profiles.

Beyond analysis of different targets, additional recent areas of growth in the field include study of bilateral lesioning. The feasibility of bilateral MRgHIFU is a point of debate in the field with some concerns coming from the experience of the clinical outcomes reported with classical stereotactic radiofrequency techniques [95]. The feasibility of staged bilateral MRgHIFU subthalamotomy for PD has been recently explored in a prospective open label series of six patients with varying time to second procedure (median 3.2 years) [39]. The MDS-UPDRS III OFF scores improved by 52.6% between baseline and 6 months after the second STN-MRgHIFU. Six months after the second procedure, the second treated side improved by 64.3%. After the second FUS-STN, four patients presented with contralateral choreic dyskinesia, which resolved after three months. Four patients developed speech disturbances, which gradually improved but remained mild in two patients at six months; and one patient experienced mild imbalance and dysphagia during the first week after treatment, which subsided after three months [39]. These findings suggested that staged bilateral STN-MRgHIFU was safe and effective for the treatment of PD, although mild but persistent speech-related adverse events were observed among some patients.

On the other hand, the concept of early application of MRgHIFu for PD has been explored in two open label studies targeting VIM and STN, respectively, showing benefits and side effect profiles similar to those observed in patients with longer disease duration [96, 97]. An international, multicenter RCT with early unilateral MRgHIFU subthalamotomy (Early Focus II) is ongoing to further clarify if additional benefit to early-stage patients may be observed (NCT06584383).

### Low Intensity Focused Ultrasound (LIFU)

While MRgHIFU is used in tissue ablation, MRgLIFU has emerged as a non-invasive neuromodulatory technique that can be used to open the BBB. Recent studies in MRgLIFU techniques have demonstrated promising safety and feasibility profiles in PD and Parkinson’s disease dementia (PDD) patients. Two phase I clinical trials demonstrated that MRgLIFU, targeting the right parieto-occipital cortex (*N* = 5) and the posterior unilateral putamen (*n* = 7) led to a transient, safe, and reversible opening of the BBB, which was associated with mild cognitive improvement [40, 41]. Meng et al. (2022) explored BBB opening with GCase enzyme delivery in four PD patients with *GBA1* mutations, demonstrating safety but also metabolic normalization in the putamen and a modest clinical improvement, as reflected by a 12% reduction in MDS-UPDRS III scores [42]. A Phase I/II study is currently ongoing to explore bilateral putaminal GCase delivery with LIFU in both *GBA1* carriers and idiopathic PDD patient (NCT05565443) (Table 1). Together, these studies highlight the potential of MRgLIFU to open the BBB as a safe, targeted strategy for drug delivery, paving the way for future therapeutic interventions. Other ongoing trials of interest include a crossover study investigating the effect of LIFU on the zona incerta ZI and VIM on tremor control (NCT06259708), and a prospective feasibility study on LIFU as an adjunct neuromodulatory treatment in PD (NCT06763692).

## Cell-Based Therapies

While the bulk of this review has covered updates in established surgical and device-aided treatment modalities, this final section will summarize updates in experimental cell-based therapies for PD that aim primarily to regenerate nigrostriatal cells and pathways. This topic has been included given the shared surgical element of this treatment and to highlight recent publications that have generated significant interest in the field.

### Human Fetal Mesencephalic Tissue

The first cell therapy studies in PD began in the late 1980s with adrenal medullary transplantation into patient striatum and were ultimately limited by safety issues as well as inconsistent and short-lived benefit. Subsequent efforts shifted focus to transplantation of stem cell sources. In the 1990s, early open-label trials were first published involving transplantation of tissue from human fetal ventral mesencephalon (hfVM) into patient striatum as a source of dopaminergic (DA) progenitor cells [98, 99]. There were encouraging results with evidence of long-term graft survival, increased striatal dopamine synthesis, and improvement in motor scores over a span of multiple years [100, 101]. However, subsequent double-blind, placebo-controlled trials demonstrated variable and less convincing efficacy. Trend towards benefit was seen in patients younger than 60 and with milder symptoms, but the studies overall failed to meet primary end points [102, 103]. They also raised concerns for suboptimal safety and tolerability, in large part due to graft-induced dyskinesias (GID), which were seen in 15–56% of patients [102, 103]. GID are defined as dyskinesias persisting despite reduction or discontinuation of levodopa. Proposed mechanisms for GID include non-homogenous delivery of transplanted cells within the putamen as well as unregulated dopamine release from serotonergic neurons included within the graft tissue [104].

In addition to the risks and questionable efficacy, hfVM transplantation has also been limited by ethical issues and logistical challenges in obtaining stable, reproducible, high-quality transplant tissue. These limitations were all further confirmed in the May 2025 publication of 3-year data from the open-label TransEuro trial. During this trial, 11 patients underwent intraputamenal transplantation of hfVM with 12 months of triple immunosuppression (cyclosporine, azathioprine, prednisolone). There was no significant effect of transplant on the primary end point of OFF UPDRS part III score. In secondary endpoints, transplanted patients had lower L-DOPA equivalent daily dose and reduced OFF time compared to controls. Notably, the trial engrafted only 11 of 20 planned individuals due to scarcity of hfVM tissue, and authors noted large variation in gestational age of available donor fetuses. From a safety standpoint, there were 7 serious adverse events (5 of which were deemed treatment-related: 2 intracerebral hemorrhages, 1 wound dehiscence, and 2 immunosuppression-related) and 3 patients developed GID (mild, nondisabling) [105].

### Stem Cells: First-In-Human Trials

Considering the challenges with hfVM transplantation, the current landscape of cell-based therapies for PD has turned dominantly towards other human pluripotent stem cell sources of DA neurons. The three most notable sources are (1) human embryonic stem cells (hESC) derived from blastocysts, (2) induced pluripotent stem cells (iPSC) commonly derived from skin fibroblasts or blood cells that are either allogenic (donor origin) or autologous (patient origin), and (3) parthenogenetic stem cells (hpSC) derived from unfertilized eggs [43, 106]. The latter type was employed in the earliest human PD stem cell trials. A Phase I study (NCT02452723), initiated in 2016 from Australia’s Cyto Therapeutics, transplanted 12 patients. Abstract publication and a company press release have reported safety as well as positive preliminary efficacy findings for ON/OFF time and quality of life [107]^,^. A Phase I/II study (NCT03119636) from the Chinese Academy of Sciences was planned to begin in 2017 for transplantation in 50 patients. No recent publications or public domain updates have been posted for either study.

In April 2025, results were published of first-in-human trials for hESC- and iPSC-derived DA cell transplantation into PD patients. Both studies met their primary endpoints of safety and tolerability. Tabar et al. (BlueRock Therapeutics) performed a Phase I trial of bilateral putamenal transplantation of an hESC-derived DA progenitor cell product (bemdaneprocel) and presented 18-month data on 12 patients (5 low dose and 7 high dose). Three serious adverse events were reported: 1 seizure attributed to surgical procedure, 1 hospitalization for COVID-19, and 1 GI hemorrhage. No adverse events were attributed to the cell product [44]. Sawamoto et al. (Kyoto University) performed a Phase I/II trial of bilateral putamenal transplantation of an allogenic (single donor) iPSC-derived combined DA progenitor cell and DA neuron product and presented 24-month data on 7 patients (3 low dose and 4 high dose) with no serious adverse events. Notably, there was 1 moderate case of dyskinesia, and 6 of 7 patients had mild worsening in ON-time dyskinesias attributed to maintenance of anti-parkinsonian medication doses throughout the trial [45]. However, neither study reported concern for GID [44, 45]. Authors attributed the lack of GID to cell purification processes which excluded serotonergic cells. Both studies utilized immunosuppression - a regimen of basiliximab and methylprednisolone perioperatively followed by prednisone and tacrolimus for one year in Tabar et al. and tacrolimus alone for 15 months in Sawamoto et al. Both studies demonstrated reasonable safety of the interventions overall without evidence of tumorigenic cell overgrowth or inflammation [44, 45].

Efficacy for each study was evaluated in secondary and exploratory outcomes. In the Tabar et al. hESC trial, mean MDS-UPDRS part III OFF motor scores improved by 8.6 points in the low-dose cohort and 23 points in the high-dose cohort; good ON time also increased by a mean 2.7 h in the high-dose group [44]. In the Sawamoto et al. iPSC trial, 6 of 7 patients had efficacy evaluation with mean improvements in OFF motor scores of 9.5 points and no apparent change in ON time [45]. Utilizing positron emission tomography, both studies demonstrated increase in ^18^Fluoro-DOPA uptake, supportive of graft cell survival and function as active DA neurons [44, 45]. Building on these proof-of-concept findings, multiple stem cell–based programs have now advanced into larger clinical trials utilizing both hESC- and iPSC-derived dopaminergic cell products.

### Stem Cells: Ongoing Clinical Trials

BlueRock Therapeutics is the furthest along of the hESC programs, now with a Phase III trial, ExPDite2 (NCT06944522) underway that plans to enroll 102 subjects. Notable other hESC-derived DA cell product clinical trials include Region Skane’s STEM-PD phase I trial (NCT05635409) in 8 patients in UK and Sweden and a Phase I/IIa study (NCT05887466) from S.Biomedics in 12 patients in Korea. The Kyoto University program discussed above is furthest along of the iPSC programs, and in collaboration with Sumitomo Pharma, a second Phase I/IIa study (NCT06482268) planned for 7 patients is now underway at the University of California, San Diego. While the Kyoto program utilized allogenic cells, other significant ongoing iPSC clinical trials are utilizing autologous iPSCs to obviate the need for immunosuppression. Of these, Aspen Neuroscience’s ASPIRO trial appears to be furthest along with a Phase I/IIa trial (NCT06344026) of autologous (skin fibroblast origin) iPSC-derived DA progenitor cell transplantation in 9 patients. Their preliminary 6-month data of 3 patients were presented in May 2025, reporting no serious adverse events, no GID, and motor score improvements [46]. A Phase I study (NCT06422208) of autologous (blood cell origin) iPSC-derived DA cell transplantation in 6 patients is also being conducted by a team at Brigham & Women’s Hospital.

In summary, stem cell therapy trials in PD began with encouraging initial results of hfVM transplantation more than 30 years ago. The field has thus turned dominantly toward human pluripotent stem cell sources of DA neurons as hfVM trials raised concerns regarding variable efficacy, GID, and logistical/ethical issues. The most notable and recent of such trials investigated transplantation of hESC- and iPSC-derived DA cell products and demonstrated favorable phase I/II safety profiles and very encouraging preliminary efficacy data. Ongoing and planned studies seek to refine techniques for cell product development and transplantation as well as more vigorously test stem cell transplantation outcomes in larger and more advanced phase trials (Table 1).

## Health System and Access Barriers To Advanced Parkinson’s Disease Therapies

Despite clear clinical benefits, real-world adoption of these advanced therapies for PD may remain limited. Across some high-income countries, DBS consistently emerges as the most cost-effective device-aided therapy, whereas LCIG and apomorphine infusion are associated with higher long-term costs driven by ongoing medication and pump requirements, despite comparable symptomatic benefit [108–110]. MRgFUS appears cost‑effective vs. medical therapy and competitive with DBS for tremor‑dominant PD in modeling studies [111]. However, comparative economic data between DBS, LCIG and apomorphine infusion are still sparse [108, 112], while cost-effectiveness analyses including newer subcutaneous levodopa infusion systems are awaited. Currently, access to these interventional therapies is obviously not universal, with socioeconomically disadvantaged patients being disproportionately, negatively affected. Limitation of access is further compounded by complex referral pathways, limited provider awareness, and heterogeneous reimbursement and regulatory frameworks [113–115]. Addressing these barriers will require expanded specialist training, coordinated policy efforts, and cost-reduction strategies to promote more equitable access to advanced PD care.

## Conclusions

Advances in interventional therapies are expanding the therapeutic repertoire for PD patients. Regulatory approvals for subcutaneous administration of levodopa-based therapies, adaptive DBS systems and expanded MRgHIFU targets reflect a maturing therapeutic landscape and the readiness for broader clinical adoption. Optimization and standardization of biomarkers for aDBS, assessing the disease-modifying potential and long-term outcome data of early DBS, MRgFUS and cell-based therapies are some of the remaining critical gaps. Comparative research across these various advanced therapies will also be crucial to guide clinical decision-making in patient selection to maximize clinical benefit.

## Key References

- Soileau MJ, Aldred J, Budur K, Fisseha N, Fung VS, Jeong A, et al. Safety and efficacy of continuous subcutaneous foslevodopa-foscarbidopa in patients with advanced Parkinson’s disease: a randomised, double-blind, active-controlled, phase 3 trial. Lancet Neurol. 2022 Dec;21(12):1099–109.
  - ○ First phase 3 double-blind active-controlled trial of foslevodopa/foscarbidopa subcutaneous infusion, proving efficacy and safety.
- Katzenschlager R, Poewe W, Rascol O, Trenkwalder C, Deuschl G, Chaudhuri KR, et al. Apomorphine subcutaneous infusion in patients with Parkinson’s disease with persistent motor fluctuations (TOLEDO): a multicentre, double-blind, randomised, placebo-controlled trial. Lancet Neurol. 2018 Sep;17(9):749–59.
  - ○ Landmark double-blind RCT of apomorphine infusion, establishing its evidence base
- Torres V, Del Giudice K, Roldán P, Rumià J, Muñoz E, Cámara A, et al. Image-guided programming deep brain stimulation improves clinical outcomes in patients with Parkinson’s disease. npj Park Dis. 2024 Jan 27;10(1):29.
  - ○ Prospective single-center study providing clinical evidence that image-guided DBS programming leads to significantly better outcomes compared to conventional programming
- Rigon L, Bove F, Izzo A, Montano N, Brusa L, Cerroni R, et al. Concordance between imaging and clinical based STN-DBS programming improves motor outcomes of directional stimulation in Parkinson’s disease. J Park Dis. 2025 Mar 16;15(2):409–20.
  - ○ Retrospective observational cohort study showing improved motor outcomes through combined image-clinical programming for directional DBS compared to standard clinical-only programming
- Oehrn CR, Cernera S, Hammer LH, Shcherbakova M, Yao J, Hahn A, et al. Chronic adaptive deep brain stimulation versus conventional stimulation in Parkinson’s disease: a blinded randomized feasibility trial. Nat Med. 2024 Nov 19;30(11):3345–56.
  - ○ First blinded randomized trial comparing adaptive vs conventional DBS, showing feasibility and benefit.
- Busch JL, Kaplan J, Habets JGV, Feldmann LK, Roediger J, Köhler RM, et al. Single threshold adaptive deep brain stimulation in Parkinson’s disease depends on parameter selection, movement state and controllability of subthalamic beta activity. Brain Stimul. 2024 Jan;17(1):125–33.
  - ○ In-depth examination of adaptive DBS thresholds and beta dynamics, informing algorithm design
- Gallay MN, Moser D, Rossi F, Magara AE, Strasser M, Bühler R, et al. MRgFUS Pallidothalamic Tractotomy for Chronic Therapy-Resistant Parkinson’s Disease in 51 Consecutive Patients: Single Center Experience. Front Surg. 2020 Jan 14;6.
  - ○ Single center experience of tract-targeting approach with MRgFUS, providing strong observational evidence that supports safety and sustained benefit in a real-world cohort.
- Krishna V, Fishman PS, Eisenberg HM, Kaplitt M, Baltuch G, Chang JW, et al. Trial of Globus Pallidus Focused Ultrasound Ablation in Parkinson’s Disease. N Engl J Med. 2023 Feb 23;388(8):683–93.
  - ○ First double-blind, randomized, controlled trial of unilateral GPi-MRgFUS, achieving clinically meaningful benefit in PD patients with motor complications, extending FUS beyond tremor and thalamic targets
- Martínez-Fernández R, Máñez-Miró JU, Rodríguez-Rojas R, del Álamo M, Shah BB, Hernández-Fernández F, et al. Randomized Trial of Focused Ultrasound Subthalamotomy for Parkinson’s Disease. N Engl J Med. 2020 Dec 24;383(26):2501–13.
  - ○ First double-blind, randomized, controlled trial of unilateral STN-MRgFUS, showing that STN lesioning with FUS is effective.
- Isaacson SH, Espay AJ, Pahwa R, Agarwal P, Shill HA, Hui J, et al. Continuous, subcutaneous apomorphine infusion for Parkinson disease motor fluctuations: Results from the phase 3, long-term, open-label United States InfusON study. J Park Dis. 2025 Mar 29;15(2):361–73.
  - ○ First large U.S. phase 3, long-term data on continuous subcutaneous apomorphine infusion, showing safety/efficacy in motor fluctuations; key for the FDA approval.
- Gharabaghi A, Groppa S, Navas-Garcia M, Schnitzler A, Muñoz-Delgado L, Marshall VL, et al. Accelerated symptom improvement in Parkinson’s disease via remote internet-based optimization of deep brain stimulation therapy: a randomized controlled multicenter trial. Commun Med. 2025 Jan 31;5(1):31.
  - ○ First RCT on remote DBS optimization, showing remote DBS programming is feasible and improves outcomes.
- Hacker ML, Rajamani N, Neudorfer C, Hollunder B, Oxenford S, Li N, et al. Connectivity Profile for Subthalamic Nucleus Deep Brain Stimulation in Early-Stage Parkinson Disease. Ann Neurol. 2023 Aug 5;94(2):271–84.
  - ○ Study showing defined connectivity profile of STN DBS in early PD patients, supporting neuroanatomical and network-guided targeting and rationale for early intervention trials.
- Barker RA, Lao-Kaim NP, Guzman NV, Athauda D, Bjartmarz H, Björklund A, et al. The TransEuro open-label trial of human fetal ventral mesencephalic transplantation in patients with moderate Parkinson’s disease. Nat Biotechnol. 2025 May 2.
  - ○ Long-term, multicenter data in a well-defined cohort of moderate PD patients, addressing safety, feasibility, and clinical outcomes.
- Tabar V, Sarva H, Lozano AM, Fasano A, Kalia SK, Yu KKH, et al. Phase I trial of hES cell-derived dopaminergic neurons for Parkinson’s disease. Nature. 2025 May 22;641(8064):978–83.
  - ○ First-in-human hESC-derived dopaminergic neuron grafts, milestone for stem-cell–based PD therapy.
- Sawamoto N, Doi D, Nakanishi E, Sawamura M, Kikuchi T, Yamakado H, et al. Phase I/II trial of iPS-cell-derived dopaminergic cells for Parkinson’s disease. Nature. 2025 May 22;641(8064):971–7.
  - ○ Early-phase iPSC-derived dopaminergic cell trial, demonstrating feasibility and translational progress.

## Data Availability

No datasets were generated or analysed during the current study.

## References

[1] Zulli C, Sica M, De Micco R, Del Petre A, Amato MR, Tessitore A, et al. Continuous intra jejunal infusion of levodopa-carbidopa intestinal gel by jejunal extension tube placement through percutaneous endoscopic gastrostomy for patients with advanced Parkinson’s disease: a preliminary study. Eur Rev Med Pharmacol Sci. 2016;20(11):2413–7. 27338069

[2] Fernandez HH, Standaert DG, Hauser RA, Lang AE, Fung VSC, Klostermann F, et al. Levodopa-carbidopa intestinal gel in advanced parkinson’s disease: final 12‐month, open‐label results. Mov Disord. 2015;30(4):500–9. 25545465 10.1002/mds.26123 PMC4674978

[3] Olanow CW, Kieburtz K, Odin P, Espay AJ, Standaert DG, Fernandez HH, et al. Continuous intrajejunal infusion of levodopa-carbidopa intestinal gel for patients with advanced Parkinson’s disease: a randomised, controlled, double-blind, double-dummy study. Lancet Neurol. 2014;13(2):141–9. 24361112 10.1016/S1474-4422(13)70293-X PMC4643396

[4] Slevin JT, Fernandez HH, Zadikoff C, Hall C, Eaton S, Dubow J, et al. Long-Term safety and maintenance of efficacy of Levodopa-Carbidopa intestinal gel: an Open-Label extension of the Double-Blind pivotal study in advanced parkinson’s disease patients. J Park Dis. 2015;5(1):165–74. 10.3233/JPD-140456 25588353

[5] Standaert DG, Rodriguez RL, Slevin JT, Lobatz M, Eaton S, Chatamra K, et al. Effect of Levodopa-carbidopa intestinal gel on Non‐motor symptoms in patients with advanced parkinson’s disease. Mov Disord Clin Pract. 2017;4(6):829–37. 29242809 10.1002/mdc3.12526 PMC5724683

[6] Antonini A, Poewe W, Chaudhuri KR, Jech R, Pickut B, Pirtošek Z, et al. Levodopa-carbidopa intestinal gel in advanced parkinson’s: final results of the GLORIA registry. Parkinsonism Relat Disord. 2017;45:13–20. 29037498 10.1016/j.parkreldis.2017.09.018

[7] Chaudhuri KR, Kovács N, Pontieri FE, Aldred J, Bourgeois P, Davis TL, et al. Levodopa carbidopa intestinal gel in advanced parkinson’s disease: DUOGLOBE final 3-Year results. J Park Dis. 2023;13(5):769–83. 10.3233/JPD-225105 PMC10473130 37302039

[8] Jost WH. A novel treatment option for intrajejunal Levodopa administration. Expert Rev Neurother. 2023;23(1):9–13. 36723452 10.1080/14737175.2023.2176222

[9] Öthman M, Nyholm D. A 4-year follow‐up of levodopa‐entacapone‐carbidopa intestinal gel treatment in parkinson’s disease. Mov Disord Clin Pract. 2024;11(12):1609–12. 39445919 10.1002/mdc3.14240 PMC11647987

[10] Weiss D, Jost WH, Szász JA, Pirtošek Z, Milanov I, Tomantschger V et al. Levodopa–entacapone–carbidopa intrajejunal infusion in advanced Parkinson’s disease – interim analysis of the ELEGANCE study. Mov Disord Clin Pract. 2025;12(8):1075–1085. 10.1002/mdc3.70046 PMC12371452 40129359

[11] Rosebraugh M, Voight EA, Moussa EM, Jameel F, Lou X, Zhang GGZ, et al. Foslevodopa/Foscarbidopa: A new subcutaneous treatment for parkinson’s disease. Ann Neurol. 2021;90(1):52–61. 33772855 10.1002/ana.26073 PMC8251848

[12] Rosebraugh M, Liu W, Neenan M, Facheris MF. Foslevodopa/Foscarbidopa is well tolerated and maintains stable levodopa and carbidopa exposure following subcutaneous infusion. J Parkinsons Dis. 2021;11(4):1695–702. 34366380 10.3233/JPD-212813 PMC8609688

[13] Soileau MJ, Aldred J, Budur K, Fisseha N, Fung VS, Jeong A, et al. Safety and efficacy of continuous subcutaneous foslevodopa-foscarbidopa in patients with advanced Parkinson’s disease: a randomised, double-blind, active-controlled, phase 3 trial. Lancet Neurol. 2022;21(12):1099–109. 36402160 10.1016/S1474-4422(22)00400-8

[14] Aldred J, Freire-Alvarez E, Amelin AV, Antonini A, Bergmans B, Bergquist F, et al. Correction: Continuous subcutaneous Foslevodopa/Foscarbidopa in parkinson’s disease: safety and efficacy results from a 12-Month, Single-Arm, Open-Label, phase 3 study. Neurol Ther. 2023;12(6):1959–60. 37817017 10.1007/s40120-023-00554-w PMC10630263

[15] Koeglsperger T, Berberovic E, Dresel C, Haferkamp S, Kassubek J, Müller R et al. Real-world experience with continuous subcutaneous foslevodopa/foscarbidopa infusion: insights and recommendations. J Neural Transm. 2025;133(2):347–359. 10.1007/s00702-025-02911-5 PMC12855247 40121314

[16] Kukkle PL, Garg D, Merello M. Continuous subcutaneous infusion delivery of apomorphine in parkinson’s disease: a systematic review. Mov Disord Clin Pract. 2023;10(9):1253–67. 37772305 10.1002/mdc3.13810 PMC10525070

[17] Katzenschlager R, Poewe W, Rascol O, Trenkwalder C, Deuschl G, Chaudhuri KR, et al. Apomorphine subcutaneous infusion in patients with Parkinson’s disease with persistent motor fluctuations (TOLEDO): a multicentre, double-blind, randomised, placebo-controlled trial. Lancet Neurol. 2018;17(9):749–59. 30055903 10.1016/S1474-4422(18)30239-4

[18] Katzenschlager R, Poewe W, Rascol O, Trenkwalder C, Deuschl G, Chaudhuri KR, et al. Long-term safety and efficacy of apomorphine infusion in Parkinson’s disease patients with persistent motor fluctuations: results of the open-label phase of the TOLEDO study. Parkinsonism Relat Disord. 2021;83:79–85. 33486139 10.1016/j.parkreldis.2020.12.024

[19] Isaacson SH, Espay AJ, Pahwa R, Agarwal P, Shill HA, Hui J, et al. Continuous, subcutaneous apomorphine infusion for Parkinson disease motor fluctuations: results from the phase 3, long-term, open-label united States infuson study. J Park Dis. 2025;15(2):361–73. 10.1177/1877718X241310727 PMC13347436 39973510

[20] De Cock VC, Dodet P, Leu-Semenescu S, Aerts C, Castelnovo G, Abril B, et al. Safety and efficacy of subcutaneous night-time only apomorphine infusion to treat insomnia in patients with Parkinson’s disease (APOMORPHEE): a multicentre, randomised, controlled, double-blind crossover study. Lancet Neurol. 2022;21(5):428–37. 35429481 10.1016/S1474-4422(22)00085-0

[21] Benabid AL, Pollak P, Gao D, Hoffmann D, Limousin P, Gay E, et al. Chronic electrical stimulation of the ventralis intermedius nucleus of the thalamus as a treatment of movement disorders. J Neurosurg. 1996;84(2):203–14. 8592222 10.3171/jns.1996.84.2.0203

[22] Limousin P, Krack P, Pollak P, Benazzouz A, Ardouin C, Hoffmann D, et al. Electrical stimulation of the subthalamic nucleus in advanced parkinson’s disease. N Engl J Med. 1998;339(16):1105–11. 9770557 10.1056/NEJM199810153391603

[23] Deuschl G, Schade-Brittinger C, Krack P, Volkmann J, Schäfer H, Bötzel K, et al. A randomized trial of Deep-Brain stimulation for parkinson’s disease. N Engl J Med. 2006;355(9):896–908. 16943402 10.1056/NEJMoa060281

[24] Rodriguez-Oroz MC, Obeso JA, Lang AE, Houeto JL, Pollak P, Rehncrona S, et al. Bilateral deep brain stimulation in Parkinson’s disease: a multicentre study with 4 years follow-up. Brain. 2005;128(10):2240–9. 15975946 10.1093/brain/awh571

[25] Isaias IU, Caffi L, Borellini L, Ampollini AM, Locatelli M, Pezzoli G et al. Case report: improvement of gait with adaptive deep brain stimulation in a patient with Parkinson’s disease. Front Bioeng Biotechnol. 2024;12:1428189. 10.3389/fbioe.2024.1428189 PMC11423205 39323762

[26] Cernera S, Oehrn CR, Hammer LH, Shcherbakova M, Yao J, Hahn A, et al. Sustained clinical benefit of adaptive deep brain stimulation in parkinson’s disease using gamma oscillations: A case report. Mov Disord. 2025;40(2):345–50. 39629629 10.1002/mds.30076 PMC11835528

[27] Caffi L, Romito LM, Palmisano C, Aloia V, Arlotti M, Rossi L, et al. Adaptive vs. Conventional deep brain stimulation: One-Year subthalamic recordings and clinical monitoring in a patient with parkinson’s disease. Bioengineering. 2024;11(10):990. 39451366 10.3390/bioengineering11100990 PMC11504236

[28] Tinkhauser G, Pogosyan A, Tan H, Herz DM, Kühn AA, Brown P. Beta burst dynamics in Parkinson’s disease OFF and ON dopaminergic medication. Brain. 2017;140(11):2968–81. 29053865 10.1093/brain/awx252 PMC5667742

[29] Feldmann LK, Lofredi R, Neumann W-J, Al-Fatly B, Roediger J, Bahners BH, et al. Toward therapeutic electrophysiology: beta-band suppression as a biomarker in chronic local field potential recordings. Npj Park Dis. 2022;8(1):44. 10.1038/s41531-022-00301-2 PMC9018912 35440571

[30] Stanslaski S, Summers RLS, Tonder L, Tan Y, Case M, Raike RS, et al. Sensing data and methodology from the adaptive DBS algorithm for personalized therapy in parkinson’s disease (ADAPT-PD) clinical trial. Npj Park Dis. 2024;10(1):174. 10.1038/s41531-024-00772-5 PMC11408616 39289373

[31] Alva L, Bernasconi E, Torrecillos F, Fischer P, Averna A, Bange M, et al. Clinical neurophysiological interrogation of motor slowing: A critical step towards tuning adaptive deep brain stimulation. Clin Neurophysiol. 2023;152:43–56. 37285747 10.1016/j.clinph.2023.04.013 PMC7615935

[32] Busch JL, Kaplan J, Habets JGV, Feldmann LK, Roediger J, Köhler RM, et al. Single threshold adaptive deep brain stimulation in parkinson’s disease depends on parameter selection, movement state and controllability of subthalamic beta activity. Brain Stimul. 2024;17(1):125–33. 38266773 10.1016/j.brs.2024.01.007

[33] Averna A, Debove I, Nowacki A, Peterman K, Duchet B, Sousa M, et al. Spectral topography of the subthalamic nucleus to inform Next-Generation deep brain stimulation. Mov Disord. 2023;38(5):818–30. 36987385 10.1002/mds.29381 PMC7615852

[34] Bond AE, Shah BB, Huss DS, Dallapiazza RF, Warren A, Harrison MB et al. Safety and efficacy of focused ultrasound thalamotomy for patients with medication-refractory, tremor-dominant parkinson disease. JAMA Neurol [Internet]. 2017;74(12):1412. Available from: http://archneur.jamanetwork.com/article.aspx?doi=10.1001/jamaneurol.2017.3098 10.1001/jamaneurol.2017.3098 PMC5822192 29084313

[35] Krishna V, Fishman PS, Eisenberg HM, Kaplitt M, Baltuch G, Chang JW, et al. Trial of globus pallidus focused ultrasound ablation in parkinson’s disease. N Engl J Med. 2023;388(8):683–93. 36812432 10.1056/NEJMoa2202721

[36] Gallay MN, Moser D, Rossi F, Magara AE, Strasser M, Bühler R et al. MRgFUS pallidothalamic tractotomy for chronic therapy-resistant Parkinson’s disease in 51 consecutive patients: single center experience. Front Surg. 2020;6:76. 10.3389/fsurg.2019.00076 PMC6971056 31993437

[37] Insightec Announces FDA, Approval of Staged. Bilateral focused ultrasound treatment for Parkinson’s disease.

[38] Martínez-Fernández R, Máñez-Miró JU, Rodríguez-Rojas R, del Álamo M, Shah BB, Hernández-Fernández F, et al. Randomized trial of focused ultrasound subthalamotomy for parkinson’s disease. N Engl J Med. 2020;383(26):2501–13. 33369354 10.1056/NEJMoa2016311

[39] Martínez-Fernández R, Natera-Villalba E, Rodríguez-Rojas R, del Álamo M, Pineda-Pardo JA, Obeso I, et al. Staged bilateral MRI-Guided focused ultrasound subthalamotomy for Parkinson disease. JAMA Neurol. 2024;81(6):638. 38739377 10.1001/jamaneurol.2024.1220 PMC11165377

[40] Gasca-Salas C, Fernández-Rodríguez B, Pineda-Pardo JA, Rodríguez-Rojas R, Obeso I, Hernández-Fernández F, et al. Blood-brain barrier opening with focused ultrasound in parkinson’s disease dementia. Nat Commun. 2021;12(1):779. 33536430 10.1038/s41467-021-21022-9 PMC7859400

[41] Pineda-Pardo JA, Gasca‐Salas C, Fernández‐Rodríguez B, Rodríguez‐Rojas R, del Álamo M, Obeso I, et al. Striatal Blood–Brain barrier opening in parkinson’s disease dementia: A pilot exploratory study. Mov Disord. 2022;37(10):2057–65. 35765711 10.1002/mds.29134

[42] Meng Y, Pople CB, Huang Y, Jones RM, Ottoy J, Goubran M, et al. Putaminal Recombinant glucocerebrosidase delivery with magnetic resonance guided focused ultrasound in parkinson’s disease: A phase I study. Mov Disord. 2022;37(10):2134–9. 36089809 10.1002/mds.29190

[43] Izrael M, Chebath J, Molakandov K, Revel M. Clinical perspective on pluripotent stem cells derived cell therapies for the treatment of neurodegenerative diseases. Adv Drug Deliv Rev. 2025;218:115525. 39880333 10.1016/j.addr.2025.115525

[44] Tabar V, Sarva H, Lozano AM, Fasano A, Kalia SK, Yu KKH, et al. Phase I trial of hES cell-derived dopaminergic neurons for parkinson’s disease. Nature. 2025;641(8064):978–83. 40240592 10.1038/s41586-025-08845-y PMC12095069

[45] Sawamoto N, Doi D, Nakanishi E, Sawamura M, Kikuchi T, Yamakado H, et al. Phase I/II trial of iPS-cell-derived dopaminergic cells for parkinson’s disease. Nature. 2025;641(8064):971–7. 40240591 10.1038/s41586-025-08700-0 PMC12095070

[46] Fraint A, Larson PS, Christine CW, Phielipp NM, Houser M, Sherman SJ, et al. Safety, tolerability, and efficacy of intracranial delivery of autologous iPSC-derived dopaminergic precursors in moderate to advanced parkinson’s disease. Parkinsonism Relat Disord. 2025;134:107630.

[47] Espay AJ, Stocchi F, Pahwa R, Albanese A, Ellenbogen A, Ferreira JJ, et al. Safety and efficacy of continuous subcutaneous levodopa–carbidopa infusion (ND0612) for parkinson’s disease with motor fluctuations (BouNDless): a phase 3, randomised, double-blind, double-dummy, multicentre trial. Lancet Neurol. 2024;23(5):465–76. 38499015 10.1016/S1474-4422(24)00052-8

[48] Verhagen Metman L, Maral Mouradian M et al. 2.2 Continous dopaminergic stimulation: concept and evidence. In: Odin P, Chaudhuri K, Aquilonious S, editors. Where are we? Continuous dopaminergic stimulation for Parkinson’s disease [Internet]. Berkshire (UK): Empowering Strategic Performance Ltd; 2023. Available from: https://www.ncbi.nlm.nih.gov/books/NBK604718/39208181

[49] Rigon L, Bove F, Izzo A, Montano N, Brusa L, Cerroni R, et al. Concordance between imaging and clinical based STN-DBS programming improves motor outcomes of directional stimulation in parkinson’s disease. J Park Dis. 2025;15(2):409–20. 10.1177/1877718X241305725 PMC13347424 40091405

[50] Aubignat M, Berro A, Tir M, Lefranc M. Imaging-guided subthalamic nucleus deep brain stimulation programming for Parkinson disease. Neurol Clin Pract. 2024;14(6):e200326. 10.1212/CPJ.0000000000200326 PMC11396028 39282508

[51] Lange F, Steigerwald F, Malzacher T, Brandt GA, Odorfer TM, Roothans J et al. Reduced programming time and strong symptom control even in chronic course through imaging-based DBS programming. Front Neurol. 2021;12:785529. 10.3389/fneur.2021.785529 PMC8606823 34819915

[52] Rolland A-S, Touzet G, Carriere N, Mutez E, Kreisler A, Simonin C, et al. The use of image guided programming to improve deep brain stimulation workflows with directional leads in parkinson’s disease. J Park Dis. 2024;14(1):111–9. 10.3233/JPD-225126 PMC10836544 38189764

[53] Brandt GA, Stopic V, van der Linden C, Strelow JN, Petry-Schmelzer JN, Baldermann JC, et al. A retrospective comparison of multiple approaches to anatomically informed contact selection in subthalamic deep brain stimulation for parkinson’s disease. J Park Dis. 2024;14(3):575–87. 10.3233/JPD-230200 PMC11091589 38427498

[54] Torres V, Del Giudice K, Roldán P, Rumià J, Muñoz E, Cámara A, et al. Image-guided programming deep brain stimulation improves clinical outcomes in patients with parkinson’s disease. Npj Park Dis. 2024;10(1):29. 10.1038/s41531-024-00639-9 PMC10821897 38280901

[55] Waldthaler J, Bopp M, Kühn N, Bacara B, Keuler M, Gjorgjevski M, et al. Imaging-based programming of subthalamic nucleus deep brain stimulation in Parkinson’s disease. Brain Stimul. 2021;14(5):1109–17. 34352356 10.1016/j.brs.2021.07.064

[56] Wilkins KB, Melbourne JA, Akella P, Bronte-Stewart HM. Unraveling the complexities of programming neural adaptive deep brain stimulation in Parkinson’s disease. Front Hum Neurosci. 2023;17:1310393. 10.3389/fnhum.2023.1310393 PMC10716917 38094147

[57] Escobar Sanabria D, Aman JE, Zapata Amaya V, Johnson LA, Farooqi H, Wang J, et al. Controlling pallidal oscillations in real-time in parkinson’s disease using evoked interference deep brain stimulation (eiDBS): proof of concept in the human. Brain Stimul. 2022;15(5):1111–9. 35921960 10.1016/j.brs.2022.07.047 PMC9798539

[58] Oehrn CR, Cernera S, Hammer LH, Shcherbakova M, Yao J, Hahn A, et al. Chronic adaptive deep brain stimulation versus conventional stimulation in parkinson’s disease: a blinded randomized feasibility trial. Nat Med. 2024;30(11):3345–56. 39160351 10.1038/s41591-024-03196-z PMC11826929

[59] Olaru M, Cernera S, Hahn A, Wozny TA, Anso J, de Hemptinne C, et al. Motor network gamma oscillations in chronic home recordings predict dyskinesia in parkinson’s disease. Brain. 2024;147(6):2038–52. 38195196 10.1093/brain/awae004 PMC11146421

[60] Smyth C, Anjum MF, Ravi S, Denison T, Starr P, Little S. Adaptive deep brain stimulation for sleep stage targeting in Parkinson’s disease. Brain Stimul. 2023;16(5):1292–6. 37567463 10.1016/j.brs.2023.08.006 PMC10835741

[61] Medtronic earns U.S. FDA approval for the world’s first Adaptive deep brain stimulation system for people with Parkinson’s. Medtronic; 2025. [Internet]. Available from: https://news.medtronic.com/2025-02-24-Medtronic-earns-U-S-FDA-approval-for-the-worlds-first-Adaptive-deep-brain-stimulation-system-for-people-with-Parkinsons#:~:text=Feb24%2C.

[62] Fleming JE, Senneff S, Lowery MM. Multivariable closed-loop control of deep brain stimulation for parkinson’s disease. J Neural Eng. 2023;20(5):056029. 10.1088/1741-2552/acfbfa 37733003

[63] Sarikhani P, Ferleger B, Mitchell K, Ostrem J, Herron J, Mahmoudi B, et al. Automated deep brain stimulation programming with safety constraints for tremor suppression in patients with parkinson’s disease and essential tremor. J Neural Eng. 2022;19(4):046042. 10.1088/1741-2552/ac86a2 PMC9614806 35921806

[64] Sasaki F, Oyama G, Sekimoto S, Nuermaimaiti M, Iwamuro H, Shimo Y, et al. Closed-loop programming using external responses for deep brain stimulation in Parkinson’s disease. Parkinsonism Relat Disord. 2021;84:47–51. 33556765 10.1016/j.parkreldis.2021.01.023

[65] Esper CD, Merola A, Himes L, Patel N, Bezchlibnyk YB, Falconer D, et al. Necessity and feasibility of remote tele-programming of deep brain stimulation systems in Parkinson’s disease. Parkinsonism Relat Disord. 2022;96:38–42. 35151948 10.1016/j.parkreldis.2022.01.017

[66] Silburn P, DeBates S, Tomlinson T, Schwark J, Creek G, Patel H, et al. Rapid development of an integrated remote programming platform for neuromodulation systems through the biodesign process. Sci Rep. 2022;12(1):2269. 35145143 10.1038/s41598-022-06098-7 PMC8831590

[67] Gharabaghi A, Groppa S, Navas-Garcia M, Schnitzler A, Muñoz-Delgado L, Marshall VL, et al. Accelerated symptom improvement in parkinson’s disease via remote internet-based optimization of deep brain stimulation therapy: a randomized controlled multicenter trial. Commun Med. 2025;5(1):31. 39890864 10.1038/s43856-025-00744-7 PMC11785990

[68] Oliviero E, Schmitz-Luhn B, Mestre TA, Chandler JA. Telemedicine and implanted brain stimulation devices: a review of legal issues. Health Technol (Berl). 2024;14(2):329–38.

[69] Fischer DL, Sortwell CE. BDNF provides many routes toward STN DBS-mediated disease modification. Mov Disord. 2019;34(1):22–34. 30440081 10.1002/mds.27535 PMC6587505

[70] Mahlknecht P, Foltynie T, Limousin P, Poewe W. How does deep brain stimulation change the course of parkinson’s disease? Mov Disord. 2022;37(8):1581–92. 35560443 10.1002/mds.29052 PMC9545904

[71] Hacker ML, Turchan M, Heusinkveld LE, Currie AD, Millan SH, Molinari AL et al. Deep brain stimulation in early-stage Parkinson disease. Neurology. 2020;95(4):e393–e401. 10.1212/WNL.0000000000009946 PMC7455319 32601120

[72] Hacker ML, Meystedt JC, Turchan M, Cannard KR, Harper K, Fan R, et al. Eleven-year outcomes of deep brain stimulation in early-stage Parkinson disease. Neuromodulation Technol Neural Interface. 2023;26(2):451–8. 10.1016/j.neurom.2022.10.051 PMC10198566 36567243

[73] Dong W, Qiu C, Lu Y, Luo B, Jiang X, Chang L et al. Effect of deep brain stimulation compared with drug therapy alone on the progression of Parkinson’s disease. Front Neurosci. 2024;17:1330752. 10.3389/fnins.2023.1330752 PMC10800581 38260017

[74] Hacker ML, Rajamani N, Neudorfer C, Hollunder B, Oxenford S, Li N, et al. Connectivity profile for subthalamic nucleus deep brain stimulation in early stage Parkinson disease. Ann Neurol. 2023;94(2):271–84. 37177857 10.1002/ana.26674 PMC10846105

[75] Mazzone P, Brown P, DiLazzaro V, Stanzione P, Oliviero A, Peppe A, et al. Bilateral implantation in globus pallidus internus and in subthalamic nucleus in Parkinson’s disease. Neuromodulation Technol Neural Interface. 2005;8(1):1–6. 10.1111/j.1094-7159.2005.05214.x 22151377

[76] Mostofi A, Evans JM, Partington-Smith L, Yu K, Chen C, Silverdale MA. Outcomes from deep brain stimulation targeting subthalamic nucleus and caudal zona incerta for Parkinson’s disease. npj Park Dis [Internet]. 2019;5(1):17. Available from: https://www.nature.com/articles/s41531-019-0089-1 10.1038/s41531-019-0089-1 PMC6704060 31453317

[77] Mitchell KT, Schmidt SL, Cooney JW, Grill WM, Peters J, Rahimpour S, et al. Initial clinical outcome with bilateral, dual-target deep brain stimulation trial in Parkinson disease using Summit RC + S. Neurosurgery. 2022;91(1):132–8. 35383660 10.1227/neu.0000000000001957 PMC9514741

[78] Cummins DD, Sandoval-Pistorius SS, Cernera S, Fernandez-Gajardo R, Hammer LH, Starr PA. Physiological effects of dual target DBS in an individual with Parkinson’s disease and a sensing-enabled pulse generator. Parkinsonism Relat Disord. 2024;122:106089. 38460490 10.1016/j.parkreldis.2024.106089

[79] Schmidt SL, Chowdhury AH, Mitchell KT, Peters JJ, Gao Q, Lee H-J, et al. At home adaptive dual target deep brain stimulation in parkinson’s disease with proportional control. Brain. 2024;147(3):911–22. 38128546 10.1093/brain/awad429 PMC10907084

[80] Elias WJ, Lipsman N, Ondo WG, Ghanouni P, Kim YG, Lee W et al. A randomized trial of focused ultrasound thalamotomy for essential tremor. N Engl J Med [Internet]. 2016;375(8):730–9. Available from: http://www.nejm.org10.1056/NEJMoa1600159 10.1056/NEJMoa1600159 27557301

[81] Chua MMJ, Blitz SE, Ng PR, Segar DJ, McDannold NJ, White PJ, et al. Focused ultrasound thalamotomy for tremor in parkinson’s disease: outcomes in a Large, prospective cohort. Mov Disord. 2023;38(10):1962–7. 37539721 10.1002/mds.29569

[82] Sinai A, Nassar M, Sprecher E, Constantinescu M, Zaaroor M, Schlesinger I. Focused ultrasound thalamotomy in tremor dominant Parkinson’s disease: long-term results. J Parkinsons Dis. 2022;12(1):199–206. 34602500 10.3233/JPD-212810 PMC8842770

[83] Peters J, Maamary J, Kyle K, Olsen N, Jones L, Bolitho S, et al. Outcomes of focused ultrasound thalamotomy in tremor syndromes. Mov Disord. 2024;39(1):173–82. 37964429 10.1002/mds.29658

[84] Bruno F, Tommasino E, Pertici L, Pagliei V, Gagliardi A, Catalucci A, et al. MRgFUS thalamotomy for the treatment of tremor: evaluation of learning curve and operator’s experience impact on the procedural and clinical outcome. Acta Neurochir (Wien). 2023;165(3):727–33. 36763132 10.1007/s00701-023-05510-z PMC10006250

[85] Saporito G, Sucapane P, Ornello R, Cerone D, Bruno F, Splendiani A, et al. Cognitive outcomes after focused ultrasound thalamotomy for tremor: results from the COGNIFUS (COGNitive in Focused UltraSound) study. Parkinsonism Relat Disord. 2023;106:105230. 36470172 10.1016/j.parkreldis.2022.105230

[86] Purrer V, Pohl E, Borger V, Weiland H, Boecker H, Schmeel FC, et al. Motor and non-motor outcome in tremor dominant parkinson’s disease after MR-guided focused ultrasound thalamotomy. J Neurol. 2024;271(7):3731–42. 38822147 10.1007/s00415-024-12469-z PMC11233288

[87] Braccia A, Golfrè Andreasi N, Ghielmetti F, Aquino D, Savoldi AP, Cilia R, et al. Magnetic Resonance–Guided focused ultrasound thalamotomy in a prospective cohort of 52 patients with parkinson’s disease: A possible critical role of age and lesion volume for predicting tremor relapse. Mov Disord. 2025;40(3):478–89. 39825750 10.1002/mds.30093 PMC11926496

[88] Gallay MN, Moser D, Magara AE, Haufler F, Jeanmonod D. Bilateral MR-guided focused ultrasound pallidothalamic tractotomy for Parkinson’s disease with 1-year follow-up. Front Neurol. 2021;12:601153. 10.3389/fneur.2021.601153 PMC7900542 33633664

[89] Dalvi A, Zucker L, Chang WC, Wu P, Kaplitt M, Sarva H et al. Staged, bilateral MR-guided focused ultrasound pallidothalamic tractotomy as a treatment for movement disorders: rationale and design of a Parkinson’s disease study (P11-5.026). Neurology. 2025;104(7_Supplement_1).

[90] Chen J, Chen C, Aoh Y, Lu M, Tsai C. Stepwise dual-target magnetic resonance‐guided focused ultrasound in tremor‐dominant Parkinson disease: one‐year follow‐up. Eur J Neurol. 2024;31(12):e16468. 10.1111/ene.16468 PMC11555146 39287607

[91] Campins-Romeu M, Conde-Sardón R, Sastre-Bataller I, Morata-Martínez C, Losada-López M, León-Guijarro JL, et al. MRgFUS subthalamotomy in parkinson’s disease: an approach aimed at minimizing lesion volume. Npj Park Dis. 2024;10(1):230. 10.1038/s41531-024-00843-7 PMC11612194 39622797

[92] Armengou-Garcia L, Sanchez‐Catasus CA, Aviles‐Olmos I, Jiménez‐Huete A, Montoya‐Murillo G, Gorospe A, et al. Unilateral magnetic Resonance–Guided focused ultrasound lesion of the subthalamic nucleus in parkinson’s disease: A prospective study. Mov Disord. 2024;39(12):2230–41. 39295191 10.1002/mds.30020 PMC11657072

[93] Martínez-Fernández R, Natera-Villalba E, Máñez Miró JU, Rodriguez-Rojas R, Marta del Álamo M, Pineda-Pardo JÁ, et al. Prospective long-term follow-up of focused ultrasound unilateral subthalamotomy for Parkinson disease. Neurology. 2023;100(13):e1395–405. 36631272 10.1212/WNL.0000000000206771 PMC10065206

[94] Paschen S, Natera-Villalba E, Pineda‐Pardo JA, del Álamo M, Rodríguez‐Rojas R, Hensler J, et al. Comparative study of focused ultrasound unilateral thalamotomy and subthalamotomy for Medication‐Refractory parkinson’s disease tremor. Mov Disord. 2025;40(5):823–33. 40028918 10.1002/mds.30159 PMC12089906

[95] Rodriguez-Oroz MC, Martínez‐Fernández R, Lipsman N, Horisawa S, Moro E. Bilateral lesions in parkinson’s disease: gaps and controversies. Mov Disord. 2025;40(2):231–40. 39726415 10.1002/mds.30090 PMC11832798

[96] Golfrè Andreasi N, Cilia R, Romito LM, Bonvegna S, Straccia G, Elia AE et al. Magnetic resonance–guided focused ultrasound thalamotomy may spare dopaminergic therapy in early-stage tremor‐dominant Parkinson’s disease: a pilot study. Mov Disord [Internet]. 2022;37(11):2289–95. Available from: https://movementdisorders.onlinelibrary.wiley.com/doi/10.1002/mds.29200 10.1002/mds.29200 PMC9804690 36036203

[97] Martínez Fernández R, Natera Villalba E, Rodriguez-Rojas R, del Álamo M, Pineda-Pardo JA, Obeso I, et al. Unilateral focused ultrasound subthalamotomy in early Parkinson’s disease: a pilot study. J Neurol Neurosurg Psychiatry. 2024;95(3):206–13. 37673642 10.1136/jnnp-2023-331211

[98] Goetz CG, Olanow CW, Koller WC, Penn RD, Cahill D, Morantz R, et al. Multicenter study of autologous adrenal medullary transplantation to the corpus striatum in patients with advanced parkinson’s disease. N Engl J Med. 1989;320(6):337–41. 2643770 10.1056/NEJM198902093200601

[99] Olanow CW, Koller W, Goetz CG, Stebbins GT, Cahill DW, Gauger LL, et al. Autologous transplantation of adrenal medulla in parkinson’s disease. 18-month results. Arch Neurol. 1990;47(12):1286–9. 2252446 10.1001/archneur.1990.00530120030006

[100] Kordower JH, Freeman TB, Snow BJ, Vingerhoets FJG, Mufson EJ, Sanberg PR, et al. Neuropathological evidence of graft survival and striatal reinnervation after the transplantation of fetal mesencephalic tissue in a patient with parkinson’s disease. N Engl J Med. 1995;332(17):1118–24. 7700284 10.1056/NEJM199504273321702

[101] Piccini P, Brooks DJ, Björklund A, Gunn RN, Grasby PM, Rimoldi O, et al. Dopamine release from nigral transplants visualized in vivo in a Parkinson’s patient. Nat Neurosci. 1999;2(12):1137–40. 10570493 10.1038/16060

[102] Freed CR, Greene PE, Breeze RE, Tsai W-Y, DuMouchel W, Kao R, et al. Transplantation of embryonic dopamine neurons for severe parkinson’s disease. N Engl J Med. 2001;344(10):710–9. 11236774 10.1056/NEJM200103083441002

[103] Olanow CW, Goetz CG, Kordower JH, Stoessl AJ, Sossi V, Brin MF, et al. A double-blind controlled trial of bilateral fetal nigral transplantation in Parkinson’s disease. Ann Neurol. 2003;54(3):403–14. 12953276 10.1002/ana.10720

[104] Barker RA, Barrett J, Mason SL, Björklund A. Fetal dopaminergic transplantation trials and the future of neural grafting in parkinson’s disease. Lancet Neurol. 2013;12(1):84–91. 23237903 10.1016/S1474-4422(12)70295-8

[105] Barker RA, Lao-Kaim NP, Guzman NV, Athauda D, Bjartmarz H, Björklund A et al. The TransEuro open-label trial of human fetal ventral mesencephalic transplantation in patients with moderate Parkinson’s disease. Nat Biotechnol. 2026;44(1):70–78. 10.1038/s41587-025-02567-2 PMC12807853 40316701

[106] Paul G, Morizane A, Kirkeby A, Takahashi J, Henchcliffe C. The future: Stem cells? Current clinical trials using stem cells for dopaminergic cell replacement. In Elsevier; 2024. pp. 191–220. Available from: https://linkinghub.elsevier.com/retrieve/pii/S2666787824000097

[107] Kern R, Garitaonandia I, Gonzalez R, Sherman G, Semechkin A, Braine E et al. Results of an open label, dose escalating, phase 1 clinical trial evaluating the safety of a human neural stem cell based therapy in Parkinson’s disease (P1.8-016). Neurology [Internet]. 2019;92(15_supplement). Available from: https://www.neurology.org/doi/10.1212/WNL.92.15_supplement.P1.8-016

[108] Smilowska K, van Wamelen DJ, Pietrzykowski T, Calvano A, Rodriguez-Blazquez C, Martinez-Martin P, et al. Cost-effectiveness of device-aided therapies in Parkinson’s Disease: a structured review. J Parkinsons Dis. 2021;11(2):475–89. 33386813 10.3233/JPD-202348 PMC8150660

[109] Pürner D, Hormozi M, Weiß D, Barbe MT, Jergas H, Prell T, et al. Nationwide retrospective analysis of combinations of advanced therapies in patients with Parkinson disease. Neurology. 2023;101(21):e2078–93. 37914414 10.1212/WNL.0000000000207858 PMC10663029

[110] Nyholm D, Eggington S, Holm A. Therapies for advanced parkinson’s disease in sweden: a cost-effectiveness analysis using Real-World data. Neurol Ther. 2025;14(3):801–12. 40117084 10.1007/s40120-025-00730-0 PMC12088999

[111] Meng Y, Pople CB, Kalia SK, Kalia LV, Davidson B, Bigioni L, et al. Cost-effectiveness analysis of MR-guided focused ultrasound thalamotomy for tremor-dominant parkinson’s disease. J Neurosurg. 2021;135(1):273–8. 32764177 10.3171/2020.5.JNS20692

[112] Marsili L, Bologna M, Miyasaki JM, Colosimo C. Parkinson’s disease advanced therapies - a systematic review: more unanswered questions than guidance. Parkinsonism Relat Disord. 2021;83:132–9. 33158747 10.1016/j.parkreldis.2020.10.042

[113] Zaman MS, Ghahari S, McColl MA. Barriers to accessing healthcare services for people with parkinson’s disease: a scoping review. J Parkinsons Dis. 2021;11(4):1537–53. 34308913 10.3233/JPD-212735 PMC8609702

[114] Auffret M, Weiss D, Stocchi F, Vérin M, Jost WH. Access to device-aided therapies in advanced parkinson’s disease: navigating clinician biases, patient preference, and prognostic uncertainty. J Neural Transm. 2023;130(11):1411–32. 37436446 10.1007/s00702-023-02668-9 PMC10645670

[115] Jimenez-Shahed J, Malaty IA, Soileau M, Yan CH, Kandukuri L, Schinkel J, et al. Association of patient characteristics, social drivers of health, and geographic location on access to device-aided therapies among medicare beneficiaries with advanced Parkinson’s disease. Parkinsonism Relat Disord. 2025;133:107322. 39965428 10.1016/j.parkreldis.2025.107322

